# How I treat elderly patients with plasma cell dyscrasias

**DOI:** 10.18632/aging.101707

**Published:** 2018-12-18

**Authors:** Maria Gavriatopoulou, Despoina Fotiou, Ioannis Ntanasis-Stathopoulos, Efstathios Kastritis, Evangelos Terpos, Meletios Athanasios Dimopoulos

**Affiliations:** 1 Alexandra Hospital, Oncology Department, Department of Therapeutics, National and Kapodistrian University of Athens, 11528 Athens, Greece; * Equal contribution

**Keywords:** Multiple Myeloma, Waldenstrom Macroglobulinemia, AL amyloidosis, elderly patients, frailty

## Abstract

Plasma cell dyscrasias are a rare heterogeneous group of hematological disorders which are more prevalent in the older part of the population. The introduction of novel agents, improved understanding of disease biology and better supportive management have improved outcomes considerably and in the era of the aging population the question of how to best manage older patients with plasma cell dyscrasias has never been more relevant. Data on how to treat these patients comes mostly from subgroup analysis as they are underrepresented in clinical trials. This review will cover issues, available evidence and recommendations relevant to diagnosis and management of the older patients with Multiple Myeloma (MM), Waldenstrom Macroglobulinemia (WM) and systemic AL Amyloidosis. What will become increasingly evident is the need to develop and establish the use of disease-specific geriatric assessment (GA) tools. Frailty status assessment using GA tools and moving away from making decisions based merely on chronological age will allow setting clear treatment goals and consequently achieving an optimum balance between effectiveness and toxicity for this complex and heterogeneous group of patients.

## Introduction

Plasma cell dyscrasias are a rare heterogeneous group of disorders characterized by the expansion of monoclonal bone marrow plasma cells. Incidence increases considerably with age and the complex question of “how to best manage elderly patients with plasma cell dyscrasias” becomes even more relevant in the era of the aging population [1]. The introduction of novel agent combinations in recent years has improved overall survival (OS) and progression free survival (PFS) in patients with plasma cell dyscrasias. The older population has been however underrepresented in clinical trials and robust data to guide treatment decisions in the elderly diagnosed with plasma cell dyscrasias is lacking. In particular, the percentage of patients >75 years of age is particularly low as the majority will fail to meet inclusion criteria for clinical trial enrolment. In addition, disease-specific frailty assessment tools need to be developed and integrated in clinical practice to allow optimum management approaches that are not solely based on chronological age [2]. Frailty is a state characterized by decreased organ reserves due to “disease, lack of activity, inadequate nutritional intake, stress, and/or the physiologic changes of aging”. Chronological age, comorbidities and the patient’s performance status alone have limited quality in capturing the heterogeneity of the older patient group and measures of function are required [3,4]. Geriatric assessment (GA) has become increasingly important in oncology, their use increases treatment tolerance and completion rates and should therefore be included in the complex treatment decision making process [5]. GA tools assess the patients’ functional and global health status allowing fine tuning of management plans and avoiding over or under-treatment [6,7]. This review aims to discuss issues that are specific to the management of the heterogeneous group of older patients diagnosed with plasma cell dyscrasias. The discussion will focus on the three most common plasma cell dyscrasias, Multiple Myeloma (MM), Waldenstrom Macroglobulinemia (WM) and systemic AL Amyloidosis. What will become increasingly evident is the need for frailty assessment tools, the complexities of defining frailty within the context of each disease, the need of having clear treatment goals that can guide clinical practice (balance between treatment effectiveness and toxicity) and the lack of evidence from clinical trials specific to this patient population.

## MULTIPLE MYELOMA

Median age at diagnosis of patients with MM is 70 years and about 34-40% of patients with MM are older than 75 years [8,9]. Outcomes have considerably improved over the last years for patients with newly diagnosed MM (NDMM) due to better understanding of disease biology, improvements in supportive care but mostly due to the addition of novel agents like proteasome inhibitors (PI), immunomodulatory drugs (IMiDs) and monoclonal antibodies (MoAbs) in the MM armamentarium [10,11]. The improvement in PFS and OS have been reflected in the older cohort of patients as well [12] but the added benefit has been less pronounced [11–14]. Survival is poorer for patients with NDMM over the age of 70 and the risk of early death is twice as high [15–18]. In a recent analysis of 827 consecutive NDMM patients, median survival in the 110 patients who were ≥80 years old was 22 months and early mortality within 2 months post diagnosis was 20% [18]. The use of less effective drug combinations, comorbidities, less favourable disease biology, increased toxicity, lower physiological reserves and early treatment discontinuation all possibly contribute to the worse outcomes associated with increased age [19].Patients >75 years’ old who receive treatment with novel agents often have similar PFS but lower OS than younger patients which can be partly explained by the effect of first line toxicities in the choice of second line treatment [20].

The challenge is to develop sensitive tools that are clinically validated to assess frailty in the heterogeneous elderly population and to move away from making clinical decisions based on chronological age and performance status [21]. Increasingly treatment decisions based on physiological age and geriatric assessments are being incorporated in clinical practice. Frail patients however continue to be underrepresented in clinical trials [22]. Frailty tools need to be used to set treatment goals and clinical trials tailored to the frailty status of the patient [23,24].

### Initial evaluation of the MM patient

Disease evaluation in MM dose not change with age and diagnosis of symptomatic disease is based on the International Myeloma Working Group (IMWG) 2014 criteria [25]. Determining whether the cause of end-organ damage is secondary to the effects of the malignant plasma cell clone rather than inter-current illness, physiological decline of organ function or comorbidities may however confound the evaluation and can be a challenge [26]. The utility of the International Stating System (ISS) in risk stratifying older patients with MM has also been questioned by some authors as beta-2-microglobulin can be increased with impaired renal function and serum albumin which can be lower secondary to malnutrition [27,28]. The Revised-International Staging System however incorporates also high risk cytogenetics, was developed using 4000 patients, one-third was over 65 years old and its prognostic value remained independent of age [29].

### Geriatric assessment tools (GCAs) in patients with Multiple Myeloma (Table 1)

Geriatric assessment (GA) is time and manpower consuming and a challenge to incorporate it in everyday practice. A more targeted and disease specific approach that uses a limited number of indicators to assess frailty is required. In the field of MM a number of frailty scoring systems have been developed [21].

#### IMWG- frailty index:

The IMWG developed in 2015 an additive scoring system (0-5) that assesses age, comorbidities and functional conditions assessed by the Charlson Comorbidity Index (CCI), Katz Activities of Daily Living (ADL) and Lawton’s Instrumental Activities of Daily Living (IADL). It categorizes patients with MM at diagnosis as fit, intermediate and frail [30]. The score is available online (http://www.myeloma frailtyscorecalculator.net/). It should be noted that age >80 highly drives the score and that it has not been validated in “real world” myeloma patients [18].

#### R-MCI frailty score:

A German cohort of 801 consecutive NDMM patients was used to develop the revised myeloma comorbidity index (R-MCI). Multivariate analysis determined impaired lung and renal function, the Karnofsky Performance status (KPS), frailty and age as highly significant for OS and these were combined to form three categories; fit (R-MCI 1-3), intermediate-fit (R-MCI 4-6) and frail (R-MCI 7-9). A web-based application is also available. (www.myeloma comorbidityindex.org)

Another group developed the Mayo frailty staging which uses the NTproBNP as an additive biomarker of frailty as it predicts survival independent of age and performance status [21,31]. Imaging techniques are also becoming of value in determining patient frailty. Recent data from a small study demonstrated a relationship between low subcutaneous adipose tissue index and poor overall survival [32]. Ongoing clinical trials are designed to tailor treatment to the frailty status of the patient.

### How we treat elderly patients with MM

### Newly diagnosed patients

Multiple trials have demonstrated the superior PFS and OS with Melphalan-Prednisone-Thalidomide (MPT) or bortezomib-melphalan-prednisone (VMP) over Melphalan and Prednisone (MP) alone. Survival benefit has been demonstrated across subgroups including patients ≥ 75 years old despite an increased incidence of grade 3 toxicities and treatment discontinuation, particularly in older patients [33–36]. Continuous Revlimid-dexamethasone (Rd) was shown to be superior to Rd for 18 cycles and MPT for 12 cycles in the FIRST trial. Carfilzomib (K), a second generation PI, was compared to bortezomib in the CLARION study (K-MP vs VMP) but PFS was comparable and AEs ≥ grade 3 (hypertension, acute renal and cardiac failure) and the number of deaths were higher in the KMP arm. Given its toxicity profile and the twice weekly treatment regimen carfilzomib can be a challenging treatment option for elderly patients [37]. The phase 3 SWOG-S0777 study demonstrated a significantly improved PFS and median OS for patients who receive bortezomib/revlimid/dexamethasone (VRd) over Rd, the benefit remained for the ≥ 65 years old cohort and toxicity profile of VRd was worse but was considered acceptable [38]. It should be noted that patients enrolled were not necessarily transplant ineligible and age was not a stratification factor. The monoclonal antibody (anti-CD38) daratumumab was assessed in non-transplant eligible > 65 years patients in the ALCYONE trial in combination with VMP (dara-VMP vs VMP) followed by daratumumab maintenance. PFS was significantly improved and the advantage was also evident for the ≥ 75 years’ old cohort [39]. The goal of maintenance post induction is to retain and further deepen the response achieved. Prolonged treatment is however associated with toxicity and can adversely affect quality of life particularly in the elderly population. So far clinical trials have shown that maintenance improves PFS but not OS in transplant-ineligible patients although this is even less clear for patients over 75 years of age [40–42].

The results of ongoing trials that aim to address treatment options specifically in frail patients and make use of GA tools are much awaited but more trials need to be designed. The ongoing TOURMALINE-MM4 trial (NCT02312258) compares oral Ixazomib versus placebo as a 2 year maintenance option in transplant ineligible patients. Another on-going phase III trial aims to address the role of ASCT in older patients and compares Rd plus/minus intensification by high-dose melphalan in patients aged 60-75 years who are fit. (NCT01090089) A dose reduced Rd schedule versus the standard Rd schedule in NDMM is being compared in a phase III trial in patients >65 years who are considered unfit and unsuitable based on the investigator’s opinion to receive approved first line treatments. Risk stratification includes the use of GA tools (NCT02215980).

### Recommendations for treatment of the elderly at diagnosis

Randomized phase 3 trials have established that the addition of novel agents to the MP backbone improves outcomes for older MM patients but other independent trials have failed to demonstrated this advantage particularly in frail patients [43]. An optimal balance between treatment efficacy and toxicity must be achieved as higher rates of AEs might translate into higher discontinuation rates and inferior survival benefits [44,45]. IMWG-frailty index and the R-MCI are both recommended tools for the identification of fit, intermediate and frail patient [21]. Based on this categorization one can adapt treatment goals and select less intensive or dose-reduced treatment schedules as appropriate. According to the recent EMN guidelines for patients categorized as fit, treatment efficacy and deep remission (defined as complete response (CR) or minimal residual disease (MRD) negativity) should be the priority. They should receive full-dose therapy including VMP, Rd or VRD. In intermediate-fitness patients one should aim to achieve a balance between safety and efficacy by using doublets and or even low dose triplets. Finally, in frail patients doing no harm and preserving quality of life should be prioritized and doublet combinations at lower doses might be required. (Table 1) In terms of maintenance, trials have demonstrated a benefit in PFS but not an OS advantage in transplant-ineligible patients although time to next treatment is prolonged.

**Table 1:** Frailty status definition and treatment goals, treatment options and dose adjustments based on frailty status in NDMM elderly patients. Adapted from Larocca et al 2018.

	**FIT**	**INTERMEDIATE**	**FRAIL**
IMWG-frailty index score	0	1	2-5
	CCI 2 :1 IADL <5: 1 ADL <4: 1 Age 76-80: 1, >80:2		
**Revised myeloma comorbidity index (R-MCI)**	0-3	4-6	7-9
	Age 60-69 KPS: 80-90%: 2, <70%: 3 Renal disease: eGFR <60:1 Lung disease: moderate/severe:1 Frailty: moderate or severe:1 ± cytogenetic unfavourable: 1		
MAYO FRAILTY INDEX	0 Age 70: 1 ECOG PS 2 :1 NT-proBNP 300 mg/L	1 (Stage I) 2 (Stage II)	3
**Goal of Treatment**	*Efficacy: deep response*	*Balance efficacy and toxicity*	*Conservative approach, low toxicity*
**Treatment Options**	Full dose therapy ASCT Triplet regimens: VMP, VRD doublet regimens: Rd	Full or reduced dose therapy Doublet regimens Rd Vd Reduced-dose triplet	Reduced dose therapy Reduced dose doublet regimens: Rd, Vd Palliative + supportive care
**Dosing Regimens – dose Levels**			
	0	-1	-2
Dexamethasone	40 mg d1,8,15,22 in 28 day cycle	20 mg d1,8,15,22 q28 day	10 mg d1,8,15,22 q28 day cycle
Melphalan	0.25mg/kg on days 1-4 on a 4-6 week schedule	0.18mg/kg on days 1-4 on a 4-6 week schedule	0.13mg/kg on days 1-4 on a 4-6 week schedule
Prednisone	2mg/kg days 1-4 q 4-6 weeks	1mg/kg days 1-4 q4-6 weeks	0.5mg/kg days 1-4 q 4-6 weeks
Thalidomide	100 -200 mg/day	50 -100 mg/day	50 mg qother day –qday
Lenalidomide	25mg d1-21 q28	15mg d1-21 q28d	10mg d1-21 d28d
Pomalidomide	4mg d1-21 q28 day	3mg d1-21 q28 days	2mg d1-21 q28 days
Bortezomib	1.3mg/m2 d 1,4,8,11 q 3 weeks	1.3mg/m2 d 1,8,15, 22 q 5 weeks	1.0mg/m2 d 1,8,15,22 q 5 weeks
Carfilzomib	20mg/m2 d1,2,8,9,15,16 in cycle 1 then 27mg/m2 cycle 2 every 4 weeks	20mg/m2 d1,2 then 27mg/m2 d 1,8,15 q 4 weeks	20mg/m2 d1,8,15 every 4 or 5 weeks
Ixazomib	4mg d 1,8,15 q28d	3mg d 1,8,15	2.3mg d 1,8,15
Daratumumab	16mg/kg weeks 1-8, weeks 9-24 d1+15 and week 25 onwards q 4 weeks	16mg/kg weeks 1-8, weeks 9-24 d1+15 and week 25 onwards q 4 weeks	16mg/kg weeks 1-8, weeks 9-24 d1+15 and week 25 onwards q4weeks
Elotuzumab	10mg/kg d1,8,15,11 cycles 1+2, and cycle 3 d1+15	10mg/kg d1,8,15,11 cycles 1+2, and cycle 3 d1+15	10mg/kg d1,8,15,11 cycles 1+2, and cycle 3 d1+15

NDMM: newly diagnosed multiple myeloma, ADL: Activity of Daily Living, IADL: Instrumental Activity of Daily Living,

CCI: Charlson Comorbidity Index, KPS: Karnofsky Performance Status, ECOG PS: ECOG Performance Status, ASCT:

Autologous Stem Cell Transplantation, VMP: bortezomib-melphalan-prednisone, VRD: bortezomib lenalidomidedexamethasone,

Rd lenalidomide-dexamethasone, Vd: bortezomib-dexamethasone

### Treating elderly MM patients in the relapse and/or refractory setting

Treatment at relapse can often be more challenging due to prior line toxicities, comorbidities, advancing age and aggressive patterns of relapse [46]. The percentage of patients >75 years are enrolled in clinical trials at relapse is even lower given that most of them will fail to meet inclusion criteria at this point. Evidence supports a prolonged OS in elderly relapsed and/or refractory MM (RRMM) patients who achieve a CR so the treatment goal should be achieving a deep response in fit patients [47]. On the other hand preserving QoL and minimizing treatment toxicity should be the main goal in frail patients [30]. Unfortunately data from clinical trials and on GA assessments in the RRMM patients is even more scarce [26]. The benefit in PFS seen in the carfilzomib-dexamethasone (Kd) versus Vd arm in the ENDEAVOR study and in the carfilzomib-lenalidomide-dexamethasone (KRd) versus Rd arm in the ASPIRE trial was maintained for patients ≥75 years [48–50]. In the POLLUX trial RRMM patients were randomized to receive Rd versus Daratumumab-Rd and the PFS benefit was even more pronounced ≥ 75-year-old patients, with higher rate of AEs but comparable discontinuation rates [51]. The PFS advantage also persisted for the older subgroup in the CASTOR trial which compared Dara-Vd vs Vd [52]. Finally the impressive overall response rate of 60% seen in RRMM patients who received pomalidomide-daratumumab in the phase 1 EQUULEUS study was the same across all age groups [53]. Pomalidomide (Pd) has also significant activity in heavily pre-treated RRMM patients and advantages seem to be similar for patients older and younger than 65 years but limited data is available for patients aged ≥ 75 years. In the phase 3 ELOQUENT-2 trial which led to FDA approval of Elotuzumab in combination with Rd for the RRMM, 20% of patients were ≥ 75 years old [54].

### Recommendations for treatment of elderly patients with RRMM

Due to the lack of clinical trials designed specifically for the elderly in the RRMM setting recommendations are mostly expert-opinion-based [21]. Patients who are non-PI refractory following lenalidomide can receive Kd or DaraVd. KRd is an option for patients’ sensitive to lenalidomide. Careful cardiovascular assessment prior to carfilzomib treatment initiation and close monitoring is required. Dara-Rd or Elo-Rd are recommended for patients who are bortezomib but not lenalidomide refractory. Fit patients should receive full-dose combinations. Elo-Rd or IRd are appropriate options for Intermediate-fit patients. KRd can be considered in intermediate-fit patients with no cardiac comorbidities. Dara-Rd or Dara-Vd triplets can improve effectiveness without increasing toxicity compared to their respective doublets even in frail patients. For IMiD and PI refractory fit patients Pd, Pd+cyclosphosphamide, single–agent daratumumab and inclusion in clinical trials are possible options. In double refractory frail patients low dose oral combinations of cyclophosphamide or melphalan +/- thalidomide can be tried.

### The role of autologous stem cell transplantation (ASCT) in the elderly population

Single-centre retrospective transplant registry analyses have demonstrated that ASCT is feasibly in the elderly fit MM patients [55,56]. Candidates should however meet strict selection criteria as the risk of toxicities may counteract the potential benefits [30,57–59]. The results of DSMM XIII study which assesses continuous Rd treatment vs Rd induction, tandem melphalan 140mg/m2-ASCT consolidation and R-maintenance in 60-75 year-old patients are eagerly awaited [60].

### Considerations in managing treatment toxicity and supportive care

Frailty determines to a considerable extent patient susceptibility to treatment related side-effects. Dose reductions or interruptions can jeopardize response rates, deter quality of life and affect OS. Prompt and effective toxicity management to achieve an optimal balance between toxicity and effectiveness is key to treatment success. Dosing alternations depend on the type of the AE and its grading [61,62] (Table 2).

**Table 2:** Management of drug related toxicities in the elderly patients with MM and recommended dose modifications.

**Adverse event**	**Suspected agent**	**Grading of AE**	**Management**	**Dose modification**
**Neutropenia**	Bortezomib Lenalidomide	Grade 4 or grade 2-3 with infection	G-CSF until recovery	25-50% reduction
**Anemia**	Bortezomib Lenalidomide	Grade 2-4	Erythropoetin or Darbopoetin for Hb <10g/dl	25-50% reduction
**Thrombo-cytopenia**	Bortezomib Lenalidomide	Grade 3 and 4	Drug interruptions Platelet transfusion for Grade 4 AE	25-50% reduction
**Bone disease**	None		Intravenous zolendronic acid, or pamindronate. Vertebroplasty if indicated, analgesia as appropriate	
Venous thrombo- embolism (VTE) – prophylaxis	IMiDs		≤ 1 risk factor for VTE: aspirin 100mg >1 risk factors for VTE: low molecular weight heparin at prophylactic dose	
VTE management	IMiDs		Therapeutic dose of LMWH or warfarin	Temporary drug interruption and full anticoagulation
**Neuropathy**	Thalidomide Bortezomib	**Grade 2 PN** **Grade 3 PN** **Grade 1 with pain or Gr 2** **Grade 2 with pain or Gr3** **Grade 4**	**Neurological assessment during treatment, immediate dose reductions recommended** **+** **Gabapetin, pregabalin, Acetyl-L-carnitin and alpha lipoic acid, opiods, calcium channel blockers, sodium channel blockers, serotonin reuptake inhibitors**	**50% dose reduction** **Treatment discontinuation until Gr 2** **25-50% dose reduction** **Dose interruption until Gr 1 and 50% dose reduction** **Treatment discontinuation**
**Skin toxicity**	Lenalidomide thalidomide	Grade 2 Grade 3-4	Antihistamines and steroids	50% drug reduction Interruption
**Infection**	Bortezomib Lenalidomide Thalidomide	Grade 2-4	Prophylaxis: Trimethoprim-cotrimoxazole for Pneumocystis carinii prophylacis during high dose dexamethasone. Acyclovir or valacyclovir for HVZ prophylaxis during PI-containing therapy, seasonal influenza vaccination, pneumococcal vaccination , **Haemophilus influenza vaccination**	

Adapted from Cerrato et al. [146] and Palumbo et al. [62].

#### Hematologic toxicities

Bortezomib causes cyclical neutropenia and thrombocytopenia [35,63]. Hematologic toxicities are amongst the most common AEs associated with thalidomide and lenalidomide use [64,65]. Granulocyte-colony stimulating factor therapy (G-CSF) should be used as primary or secondary prophylaxis for neutropenia and erythropoiesis-stimulating agents (ESA) can be used to manage anemia which does not respond to treatment.

#### Peripheral neuropathy (PN):

Bortezomib and thalidomide-based therapies are associated with considerable rates of PN. In frail patients PN increases risk of falls and impairs function [66]. Toxicity is cumulative and dose-dependent but reversible for bortezomib contrary to the permanent neuropathy often associated with thalidomide [67,68]. Careful monitoring and patient education are imperative. The initial dosing schedule should be guided by the patients’ frailty status and immediate dose reductions are recommended. Options for management exist have not been evaluated prospectively (Acetyl-L-carnitin, alpha lipoic acid, opioids, gabapentin, pregabalin) [61].

#### Thrombosis:

The incidence of venous thromboembolism (VTE) in MM patients ranges approximately from 8 to 22/1000-person years. The risk is disease, patient and treatment-dependent [69]. IMiDs, multi-agent chemotherapy regimens and high dose dexamethasone increase VTE risk substantially [70]. VTE risk assessment and appropriate thromboprophylaxis is imperative. IMWG guidelines recommend low dose aspirin for patients on IMiDs with none or one risk factor and low molecular weight heparin or full dose warfarin when more than one VTE risk factors is present.

#### Infections:

Disease- related immunoparesis and myelosuppression secondary to treatment regimens render MM patients at high risk of infection. The risk is higher at diagnosis and mostly associated with IMiDs [71,72]. Infection prophylaxis has been shown to decrease treatment associated morbidity in MM patients. Prophylactic antiviral medication to reduce PI associated herpes zoster infection [73,74], seasonal influenza, streptococcal pneumonia and Hemophilus influenza vaccination [61] and prophylactic trimethoprim-sulfamethoxazole are all recommended [75]. A recent abstract presentation from a phase 3 clinical trial demonstrated that prophylactic use of levofloxacin reduces febrile episodes and death in NDMM patients [76].

In the case of frail patients, decision-making capacity should also be assessed to determine whether there is need for a surrogate decision-maker [77]. Finally issues related to adherence to oral treatment and the presence of appropriate socioeconomic support also come into play [78,79].

### Commentary

The complexities of managing the patient with MM are increasing in the era of the aging population and increasing treatment options. Older patients have been up to date underrepresented in clinical trials and evidence to guide management is therefore lacking. Frailty assessment tools are being developed and should be increasingly incorporated in clinical trials and clinical practice. Management of the older patient with MM should be in all contexts tailored to the patients’ frailty status. The results of current ongoing clinical trials that incorporate tools that assess frailty and are tailored to older MM patients are eagerly awaited.

## WALDENSTROM MACROGLOBULINEMIA

Waldenstrom’s Macroglobulinemia (WM) is a rare lymphoproliferative disorder characterized by the proliferation of lymphoplasmacytic elements and the presence of monoclonal immunoglobulin M (IgM) gammopathy. It is a disease of the elderly (median age at diagnosis 63-75 years) and comes with the age-related comorbidities which complicate patient management [80,82]. It is considered an indolent yet incurable disease with median disease-specific survival of 10-11 years. Despite an increase in therapeutic options there is no precise treatment algorithm due to a paucity of comparative high quality data. Older and medically non-fit patients are underrepresented in the existing clinical trials [83]. There are no frailty scores available specifically for patients with WM and up to date clinical trials have not included any scoring systems to categorize patients as fit or unfit. The ESMO consensus proposes three different categories of fitness in the context of treatment feasibility. For an “elderly fit” patient the treatment related and unrelated AEs would be comparable to those for a young “fit” patient. In contrast the “vulnerable patient” would have higher risk of treatment related and unrelated AEs. Finally, the “terminally ill” patient has a short life expectancy and will only benefit from best supportive care [84]. There is however no tool to categorize patients and therefore frailty assessment is left to clinical judgement. In the International prognostic scoring system for WM age has significant weighting. Patients aged ≥65 years are categorized as at least intermediate risk [85]. Patients with asymptomatic disease should be closely monitored and treatment initiation should be based on the IWWM-8 consensus criteria [86]. Care is required to exclude other possible diagnoses related to comorbidities of the older patient with WM.

### Management of elderly patients with WM

### Overview of treatment options

Patients with symptomatic hyperviscosity should undergo plasmapheresis followed rapidly by cytoreductive treatment. The mainstay of treatment and backbone to most treatment combinations are anti-CD20 monoclonal antibodies (Rituximab). Rituximab monotherapy has a very safe toxicity profile but is inferior to combinations [87]. To avoid IgM flare, rituximab monotherapy is not recommended for patients with high IgM levels but is indicated for patients with WM-related immunologic disorders [88,89]. The combination of Rituximab, dexamethasone and cyclophosphamide (DRC) achieves better responses, has a favourable short and long term toxicity profile and is an option for patients with comorbidities and low tumour burden [90]. Another well-tolerated effective combination is Bendamustine+Rituximab (BR) but dose adjustments might be necessary in older medically non-fit patients due to associated myelosuppression risk [91]. Bortezomib (V) is also very active in patients with WM (subcutaneously, weekly, at 1.6mg/m^2^ dose) [92–94]. Bortezomib containing combinations are first choice in patients with hyperviscosity, high IgM levels, renal impairment, cryoglobulinemia or cold agglutinemia [95]. Carfilzomib in the relapse setting is an alternative neurotoxicity-sparing option among PI inhibitors [96,97]. More intensive chemotherapy regimens can induce high response rates but are not favoured for use in first line due to the associated significant toxicity. These include R-CHOP and nucleoside analogues (fludarabine/rituximab (FR) or fludarabine/cyclophosphamide/ rituximab FCR) or chlorambucil [97,98]. Ibrutinib (bruton’s tyrosine kinase inhibitor) is a very effective oral option in WM patients [99,100]. In the USA, FDA has granted approval for both first line and relapse. In Europe the EMA has licensed the drug for use at relapse and as first line for patients who are non-eligible for “immunochemotherapy”. The INNOVATE study compared rituximab/ibrutinib to rituximab monotherapy in pre-treated rituximab sensitive patients and in untreated patients. At 30 months PFS was 82% in the ibrutinib/rituximab arm versus 28% in the rituximab arm [101]. The advantage was also seen in previously untreated patients. Maintenance treatment with rituximab is currently not recommended due to the lack of prospective data [102]. ASCT is also an option for salvage therapy in WM in young patients with refractory disease or multiple relapses [97].

### Recommendations for treating elderly patients with WM

**Figure 1:**
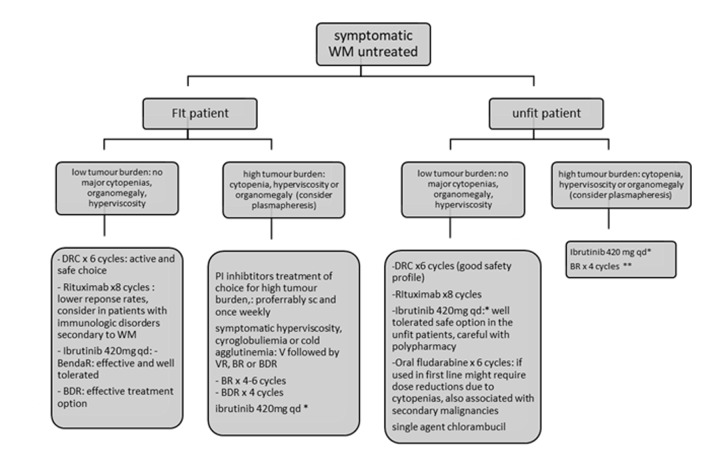
**Recommendations for the treatment of newly diagnosed patients with Waldenstrom’s Macroglobulinemia.** Figure adjusted from ESMO guidelines for WM 2018 and EMN recommendations for treatment of rare plasma cell dyscrasias. *Approved in USA by FDA for first line and only for patients unfit for immunochemotherapy in Europe by EMA. ** BR for unfit patients may require dose reductions for bendamustine and use of G-CSF and antibiotic prophylaxis. BDR: bortezomib, dexamethasone, rituximab, BR: bendamustine, rituximab; DRC: dexamethasone, rituximab, cyclophosphamide; AF: atrial fibrillation, V: bortezomib.

**Figure 2:**
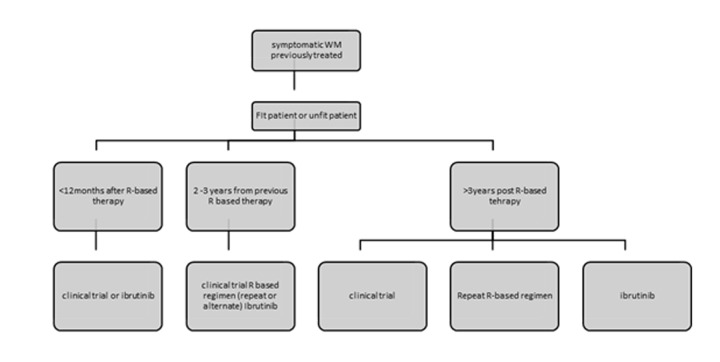
**Recommendations for the treatment of previously treated patients with Waldenstrom’s Macroglobulinemia.** Figure adjusted from ESMO guidelines for WM 2018 and EMN recommendations for treatment of rare plasma cell dyscrasias. R: rituximab.

The recent ESMO and EMN recommendations base treatment recommendations on tumour burden assessment/clinical presentation and the patient’s fitness status [95,103]. PI-combinations like bortezomib alone, BDR and bortezomib-rituximab are recommended for patients with hyperviscosity or bulky disease in fit patients. Ibrutinib is another option but responses are slower. In patients with cytopenias DRC, PI-based therapy, BR or ibrutinib are all appropriate. If neuropathy bortezomib should be avoided and for AL amyloidosis related to WM, PI-based therapy or BR are recommended [95]. For the unfit patient oral fluradarabine for 6 cycles or chlorambucil for 12 cycles, DRC for 6 cycles and rituximab monotherapy are all acceptable options if disease burden is low. VR or ibrutinib are preferred for higher tumour burdens (Figure 1). For early relapse (<1year after R-based therapy) patients should be included in a clinical trial. Ibrutinib is appropriate for early or late relapses and should be given until disease progression as drug discontinuation leads to frequent relapses. The safety profile is safe and therefore it is appropriate for medically unfit patients [100,104]. Note that ibrutinib is not recommended for patients with MYD88^WT^ disease. For relapses > 2 years post the previous R-based therapy one can repeat or alternate all the available treatment options at first line [90]. Lenalidomide alone or in combination with DRC has been used in clinical trials in heavily pre-treated populations and the combination achieved a 80% response rate and a median PFS of 24 months [105] (Figure 2).

### Commentary

The indolent nature of the disease, the reasonably safe toxicity profile of the therapeutic regimens together with appropriate reasoning on the choice of the regimen and dose adjustments can allow for effectiveness and acceptable quality of life for both fit and unfit elderly patients with WM. Median survival for younger patients exceeds 10 years. It is shorter for elderly patients but a significant proportion will die due to reasons unrelated to disease [82]. Treatment induced myelodysplasia or WM transformation to more aggressive lymphoma are commonly seen in the older patient cohorts**.** The disease does however in the elderly commonly transform or can develop to myelodysplasia [106–108]. Secondary non-hematological malignancies develop in about 18% of patients secondary to treatment toxicity [109]. It is realistic to pursue the development of more effective and at the same time minimally toxic treatment strategies [83]. Current recommendations distinguish between fit and non-fit patients but no specific tool is available to guide stratification. The success of such a pursue requires the development of disease specific frailty assessment tools and more clinical trials that test regimens in patient subgroups including the elderly.

## PRIMARY AL AMYLOIDOSIS

Immunoglobulin light chain (AL) amyloidosis is the most common type of amyloidosis in Western countries. A plasma cell clone secretes a patient specific monoclonal light chain which is amyloidogenic and causes progressive decline of vital organ function [110]. Median age of diagnosis is 62 years and the disease presents most commonly in the seventh decade of life [111]. Incidence increases with age as proteostasis progressively declines but also due to aforementioned increased incidence of associated and co-existent plasma cell dyscrasias [112,113]. Frailty in amyloidosis is associated with the type and degree of organ involvement rather than chronological age of the patient per se more so than in other hematological malignancies. The systemic nature of the disease, the rapidly progressive decline in vital organ function and the often delayed diagnosis make a large proportion of patients frail at diagnosis. Elderly patients represent however an even frailer group [114]. Older patients with AL are also underrepresented in clinical trials and GA tools are lacking. An additional complexity lays in the diagnosis of AL amyloidosis as its presentation can mimic conditions that are more prevalent in the elderly population. High clinical suspicion and appropriate diagnostic investigations are necessary to set the correct diagnosis in a timely manner.

### Clinical picture and diagnosis

The presentation of AL amyloidosis is heterogeneous and depends on organ involvement. Target organs include the heart, kidneys, soft tissues, liver, peripheral and autonomic nervous system [115]. Cardiac involvement is seen in 82% of patients and presents as restrictive cardiomyopathy. The revised Mayo staging system includes 4 stages of cardiac disease severity using cardiac biomarker assessment (NT-proBNP and cardiac troponins I or T or high sensitivity cTnT) and measurement of the free light chains (FLC) [116]. NT-proBNP can also increase in other more common cardiac conditions and due to renal impairment [117]. The value of the prognostic system has therefore been questioned in older patients [118]. Renal involvement is seen in approximately 68% of patients and presents as albuminuria and progressive decline in renal function [119]. Kidney biopsy is often required to determine the cause of albuminuria, particularly in patients with comorbidities such as chronic hypertension and diabetes. Caution is required as other types of amyloidosis are included in the differential diagnosis particularly in the elderly population. All elderly Caucasian males with cardiac involvement should have a Tc-99m 3,3-diphosphono-1,2-propanodicarboxylic acid (Tc-99m DPD) bone scan to exclude transthyretin-related cardiac amyloidosis (ATTR). About 39% of patients with ATTR will also have a plasma cell dyscrasia leading to increased chances of misdiagnosis [120,121]. Cases of hereditary amyloidosis coexisting with monoclonal gammopathy of undetermined significance and AA amyloidosis coexisting with IgM monoclonal gammopathy have also been reported [122–124]. Amyloid typing is mandatory in all the above cases using mass spectrometry, immnunoelectron microscopy or immunohistochemistry.

### How we treat elderly patients with AL amyloidosis

**Figure 3:**
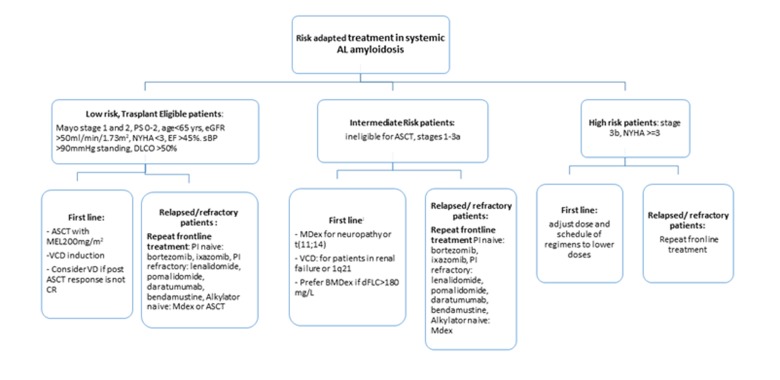
**Risk adapted treatment recommendations in systemic AL amyloidosis.** Adapted from Palladini et al 2016(115) and Gavriatopoulou et al 2018. Data mainly comes from uncontrolled trials. ASCT: autologous stem cell transplant, DLCO: lung diffusion of CO, EF: ejection fraction, MEL: melphalan, NYHA: New York Heart Association, OS: performance status by ECOG, sBP: systolic blood pressure, Stage is Mayo Clinic cardiac stage, VCD: velcade+cyclophosphamide+dexamethasone, CD: velcade+ dexamethasone, MDex: melphalan+dexamethasone, CR: complete response, PI: proteasome inhibitor, BMDex: bortezomib+ melphalan+dexamethasone.

The primary aim of therapy is to achieve a hematologic response by reducing the amyloidogenic light chain production via plasma cell clone eradication. A deep haematological response is a prerequisite for the secondary aim which is the gradual restoration of the target organ function [125]. Treatment is guided on the basis of cardiac staging and assessment of patient fitness and clinical trials for the older AL patients are lacking [126]. There are strict eligibility criteria for ASCT in patients with AL amyloidosis [114]. A cut-off at 70 years is used by most centres and patients > 65 years receive reduced conditioning [127]. Non-eligible patients will receive risk-adapted therapy. Conventional treatment is based on alkylating agents (melphalan and cyclophosphamide), PIs and IMiDs [128,129]. Retrospective studies have demonstrated up to 90% hematologic response with the triplet combination of Bortezomib-Cyclophosphamide-Dexamethasone (VCD) in first line treatment [130,131]. Interim analysis of a phase 3 clinical trial comparing melphalan-dexamethasone (MDex) to bortezomib-MDex showed higher responses with the latter. Ixazomib compared to physicians’ best choice is being assessed currently [132] and IMiDs are used mainly in the relapsed/refractory setting [133,134]. Addition of alkylating agents to IMiDs can achieve even higher responses but myelotoxicity is a concern [135]. Daratumumab monotherapy also yielded haematological responses in heavily pre-treated AL patients [136]. Contraindications to agent use include neuropathy for bortezomib, renal failure for lenalidomide. Agents that target the amyloid deposits (passive immunotherapy) with the aim of accelerating removal and restoring organ function are currently being investigated in clinical trials [137–139]. Current EMN guidelines suggest a risk-adapted stratification treatment approach which includes age (Figure 3). For low risk-transplant eligible patients VCD induction followed by MEL 200 mg/m^2^ plus bortezomib post ASCT if complete response has not been achieved. Intermediate risk patients should receive MDex or BMDex. ΜDex alone for patients with neuropathy and t(11;14) translocation and for patients with 1q21 gain or renal failure VCD [140]. Close monitoring of dFLC is required to initiate treatment at relapse without waiting for organ progression but no consensus exists on timepoint of treatment re-initiation. Lenalidomide, pomalidomide ixazomib or bendamustine are all indicated at relapse [141]. Frontline treatment can be repeated or switched to another option for refractory patients. The complex nature of the disease makes supportive management imperative to treatment-related toxicity minimization and increased tolerability. The poor physiological reserves associated with target organ dysfunction require a multidisciplinary approach that requires heart failure specialists, nephrologists, haematologists and nutrition specialists [142–145].

### Commentary

Frailty is a more complex construct in AL amyloidosis compared to other plasma cell dyscrasias owing to the nature of the disease. Frailty assessment tools are lacking and older patients are underrepresented in clinical trials. Development of GA tools will be a more difficult endeavour for this heterogeneous disease as multiple physiological parameters will need to be accurately assessed to appropriately categorize patients and set treatment goals. Clinical trials designed specifically for older or more frail patients are needed to guide evidence based medicine in these patients with poor overall outcomes. Increased awareness of the entity of AL amyloidosis and consideration in the differential diagnosis of physicians is imperative for timely diagnosis. Finally, pre-emptive supportive measures is key to maximize treatment tolerance and improve treatment outcomes.
